# Computational Modeling of Pro-inflammatory Cytokine-Enhanced Blood Coagulation

**DOI:** 10.34133/csbj.0180

**Published:** 2026-08-05

**Authors:** Geli Li, Chen Zhao, Galit H. Frydman, He Li

**Affiliations:** ^1^ School of Chemical, Materials, and Biomedical Engineering, University of Georgia, Athens, GA, USA.; ^2^ School of Pharmacy, Nanjing Medical University, Nanjing, China.; ^3^ Division of Comparative Medicine, Department of Biological Engineering, Massachusetts Institute of Technology, Cambridge, MA, USA.; ^4^ Division of Trauma, Emergency Surgery, and Surgical Critical Care, Massachusetts General Hospital, Boston, MA, USA.

## Abstract

The interplay between inflammation and coagulation is a central driver of thrombotic risk across various diseases. While mathematical models of blood coagulation are well established, there remains a critical gap in quantitative frameworks that capture inflammation-induced hypercoagulability. In this study, we develop a mathematical model that explicitly simulates the interaction between pro-inflammatory cytokines and the coagulation cascade. The model incorporates key mechanisms, including: (a) up-regulation of tissue factor by interleukin-1𝛽, interleukin-6, and tumor necrosis factor-α; (b) suppression of natural anticoagulants, namely, antithrombin III and tissue factor pathway inhibitor, by interleukin-6 and tumor necrosis factor-α; and (c) feedback amplification of pro-inflammatory cytokines by thrombin. By encoding the bidirectional feedback between inflammatory and coagulation pathways, the model captures essential features of inflammation-driven hypercoagulability and enables systematic quantification of how variability in inflammatory extent and duration result in heterogeneous thrombin generation (TG) dynamics. To evaluate its effectiveness, we integrate the model with TG assays and apply it to virtual patient cohorts representing 4 clinically distinct conditions: COVID-19, sickle cell disease, type 2 diabetes mellitus and hemophilia A. Model simulations predict that disease-specific inflammatory environments induce distinct shifts in TG dynamics. In COVID-19 and type 2 diabetes mellitus, elevated cytokine levels lead to shortened lag times and increased thrombin peak, whereas in sickle cell disease, shortened lag times are accompanied by a reduced thrombin peak. These effects are strongly modulated by both cytokine concentration and duration of exposure. These results demonstrate that the proposed computational model augments conventional TG assays by mechanistically linking inflammatory signaling to disease-specific coagulation responses. Collectively, the proposed computational framework extends conventional TG assays by considering the interplay between inflammation and coagulation, thereby providing a mechanistic exploratory platform for generating testable hypotheses regarding disease-specific thromboinflammatory regulation. This framework lays a foundation to support future clinical prediction or individualized therapeutic decision-making after validation using prospective patient-level longitudinal datasets.

## Introduction

Human blood coagulation is a tightly regulated biochemical network that maintains hemostatic balance after vascular injury [1]. Its central physiological function is to limit blood loss through rapid clot formation at sites of vascular damage, followed by controlled fibrinolytic resolution. Disruption of this balance can lead to a broad spectrum of coagulation dysregulation, ranging from hypocoagulable states caused by factor deficiency, such as hemophilia A [2], to inflammation-associated hypercoagulable states observed in diseases such as COVID-19, sickle cell disease (SCD), and type 2 diabetes mellitus (T2DM) [3–6]. Insufficient coagulation factor levels or dysregulated fibrinolysis can result in uncontrolled bleeding [7], whereas excessive production of procoagulant or prothrombotic factors can drive inappropriate clot formation and thrombosis [8]. Thus, disease-associated abnormalities in coagulation should not be viewed as a single prothrombotic phenotype, but as a continuum shaped by coagulation factor availability, endogenous anticoagulant activity, fibrinolytic regulation, and inflammatory signaling.

Inflammation–coagulation coupling is increasingly recognized as a central mechanism by which immune activation alters thrombin generation (TG) and thrombotic risk across diverse pathological conditions [9–12]. Inflammatory signaling perturbs hemostatic homeostasis by simultaneously up-regulating procoagulant pathways and weakening endogenous anticoagulant and fibrinolytic safeguards [13,14]. Pro-inflammatory cytokines, including interleukin-6 (IL-6), interleukin-1𝛽 (IL-1𝛽), and tumor necrosis factor-α (TNF-α), can induce tissue factor (TF) expression in monocytes and endothelial cells, thereby promoting TF-dependent initiation of coagulation [15–17]. In parallel, inflammatory signaling can weaken endogenous anticoagulant regulation, including pathways mediated by antithrombin III (ATIII), tissue factor pathway inhibitor (TFPI), and the protein C system, while also attenuating fibrinolysis [18]. Inflammation can also alter downstream coagulation components, such as fibrinogen, which is both a coagulation substrate and an inflammation-associated protein reported to be elevated in severe COVID-19 [19]. When excessive or sustained, this immunothrombotic response can become maladaptive, contributing to microvascular thrombosis, end-organ hypoperfusion, and organ dysfunction in settings such as sepsis, severe viral infection, and chronic inflammatory disease [3,20,21].

These interactions are dynamic, nonlinear, and strongly dependent on exposure duration. Thrombin itself can feed back into inflammatory signaling through protease-activated receptor-associated pathways, creating a bidirectional loop in which inflammation enhances coagulation and coagulation further reinforces inflammation [22,23]. In addition, inflammation enhances endothelial dysfunction and platelet activation and strengthens platelet–leukocyte and platelet–innate immune cell interactions, thereby amplifying thrombin formation through coupled cellular and plasma-protease mechanisms [24–27]. Such inflammation-driven coagulation abnormalities are widely observed in severe inflammatory diseases, including sepsis and COVID-19, where pathways linked to TF, platelets, neutrophils, and endothelial activation contribute to systemic coagulopathy and thrombotic complications [3,28,29]. However, isolated clinical measurements, such as single time-point cytokine levels, D-dimer, prothrombin time, or activated partial thromboplastin time, provide limited information about how inflammatory burden and coagulation factor variability jointly shape TG over time. This limitation motivates the use of mechanistic mathematical models that can integrate inflammatory inputs, coagulation factor dynamics, and feedback regulation within a time-resolved framework [30,31].

Current mathematical models for simulating blood coagulation can be broadly categorized into ordinary differential equation (ODE) models and partial differential equation, spatial, or flow-based models. ODE models are well suited for simulating TG in well-mixed systems because many reactions in the coagulation cascade can be represented using first-order kinetics, second-order mass-action kinetics, and enzyme-mediated mechanisms [31–39]. These models describe temporal changes in coagulation factor concentrations and have been widely used to investigate TF-initiated TG, endogenous anticoagulant regulation, and reaction-level control of clotting dynamics. In contrast, partial differential equation-based and hybrid models account for spatial transport, thrombus growth, and blood flow effects, often through convection-diffusion-reaction formulations [40–51]. Recent computational fluid dynamics and digital twin studies further highlight the importance of integrating coagulation kinetics with patient-specific flow environments when modeling thrombosis risk in vascular and cardiac settings [52–54]. In parallel, emerging patient-specific modeling approaches have attempted to infer coagulation kinetic parameters from clinical variables or hybrid neural-network–ODE frameworks, suggesting a route toward clinically informed mechanistic modeling [55,56]. Despite these advances, most established models do not explicitly integrate cytokine-mediated TF regulation, cytokine-associated suppression of endogenous anticoagulants, and thrombin-driven inflammatory feedback within a unified disease-comparative virtual patient framework.

To address this gap, we developed an ODE-based computational model that couples pro-inflammatory cytokine dynamics with a TF-initiated coagulation cascade. The model incorporates 3 key interactions: cytokine-mediated up-regulation of TF by IL-6, IL-1𝛽, and TNF-α; cytokine-associated suppression of ATIII and TFPI; and thrombin-mediated amplification of pro-inflammatory cytokine production. We applied this framework to TG-calibrated virtual patient cohorts representing COVID-19, SCD, T2DM, and hemophilia A, thereby covering both inflammation-associated hypercoagulability and factor-deficiency-driven hypocoagulability. By linking inflammatory cytokine dynamics with TG in a disease-comparative virtual patient framework, this study provides a mechanistic and hypothesis-generating approach for analyzing disease-specific thromboinflammatory responses across distinct coagulation dysregulation states.

## Data and Methods

### Overview of the modeling workflow

The study was performed in 5 steps. First, a TF-initiated coagulation module was constructed based on the mechanistic framework of Hockin et al. [36], and the TG profiles reported in the Hockin framework were used to verify that the reconstructed coagulation module correctly reproduced the expected TF-initiated coagulation dynamics. Second, an inflammation module describing IL-6, IL-1𝛽, and TNF-α dynamics was developed using reported cytokine half-lives and baseline steady-state constraints [57–59]. Third, the 2 modules were coupled through cytokine-mediated TF up-regulation, cytokine-associated ATIII and TFPI suppression, and thrombin-mediated cytokine amplification. The parameters associated with these coupling reactions were calibrated using published experimental datasets describing cytokine-induced TF regulation and thrombin-associated inflammatory signaling [60–63]. Fourth, disease-specific virtual patient cohorts were generated by Latin hypercube sampling using reported coagulation factor and cytokine ranges for COVID-19, SCD, T2DM, hemophilia A, and normal subjects. The disease-specific coagulation factor sampling ranges were calibrated using experimentally reported peak thrombin values, and the resulting calibrated ranges were subsequently evaluated using additional TG metrics, including lag time and time to peak (TTP). Finally, the calibrated virtual cohorts were used to analyze the effects of inflammatory intensity and exposure duration on TG, with model behavior quantified using TG metrics and partial rank correlation coefficients (PRCCs)-based sensitivity analysis.

### Inflammation–coagulation coupling and endogenous inhibitory feedback

**Fig. 1.**
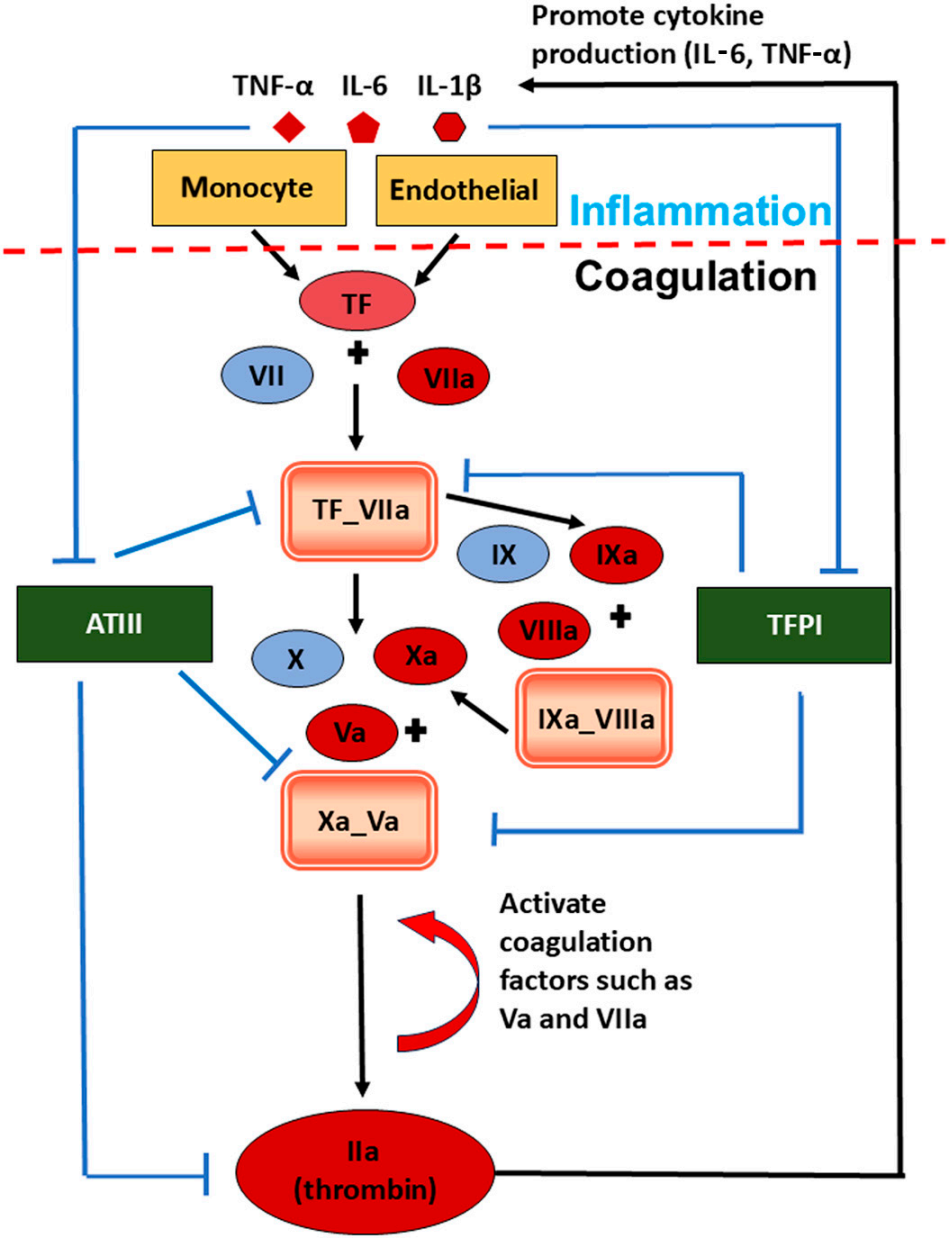
Schematic diagram of the coupled inflammation–coagulation model structure. The inflammation subsystem describes interleukin-6 (IL-6), interleukin-1𝛽 (IL-1𝛽), and tumor necrosis factor-α (TNF-α) dynamics and their effects on monocyte and endothelial cell activation. The coagulation subsystem describes tissue factor (TF)-initiated thrombin generation. Pro-inflammatory cytokines promote TF availability and attenuate endogenous anticoagulant regulation through antithrombin III (ATIII) and tissue factor pathway inhibitor (TFPI), whereas thrombin feeds back to amplify inflammatory signaling.

To explicitly represent inflammation–coagulation coupling, the model links pro-inflammatory cytokine exposure to cellular activation, TF availability, TG, and endogenous anticoagulant feedback. The final ODE system used in this study retained only species and parameters present in the curated supplementary reaction and parameter tables. As illustrated in Fig. 1, the model is organized into 2 functionally distinct but mechanistically coupled subsystems representing inflammation and coagulation. The inflammation subsystem governs the time-dependent dynamics of IL-6, IL-1𝛽, and TNF-*α*, whereas the coagulation subsystem captures TF-initiated TG.

The inflammatory component was represented through cytokine-driven activation of monocytes and endothelial cells. IL-6, IL-1𝛽, and TNF-*α* were treated as inflammatory inputs that promote conversion of resting monocytes and endothelial cells into activated states. This formulation is based on experimental evidence that inflammatory cytokines can promote TF expression primarily through monocyte and endothelial cell activation [60,61]. The activation dynamics were described as:

$$\frac{\mathrm{dM}}{\mathrm{dt}}=-k_{a,\mathrm{TNF}}^{M}{\mathrm{MC}}_{\mathrm{TNF}}-k_{a,\mathrm{IL}6}^{M}{\mathrm{MC}}_{\mathrm{IL}6}-k_{a,\mathrm{IL}1}^{M}{\mathrm{MC}}_{\mathrm{IL}1}+k_{\mathrm{da}}^{M}M_{a},$$ (1)

$$\frac{{\mathrm{dM}}_{a}}{\mathrm{dt}}=k_{a,\mathrm{TNF}}^{M}{\mathrm{MC}}_{\mathrm{TNF}}+k_{a,\mathrm{IL}6}^{M}{\mathrm{MC}}_{\mathrm{IL}6}+k_{a,\mathrm{IL}1}^{M}{\mathrm{MC}}_{\mathrm{IL}1}-k_{\mathrm{da}}^{M}M_{a},$$ (2)

$$\frac{d\mathrm{EC}}{\mathrm{dt}}=-k_{a,\mathrm{TNF}}^{\mathrm{EC}}\mathrm{EC}C_{\mathrm{TNF}}-k_{a,\mathrm{IL}6}^{\mathrm{EC}}\mathrm{EC}C_{\mathrm{IL}6}-k_{a,\mathrm{IL}1}^{\mathrm{EC}}\mathrm{EC}C_{\mathrm{IL}1}+k_{\mathrm{da}}^{\mathrm{EC}}{\mathrm{EC}}_{a},$$ (3)

$$\frac{d{\mathrm{EC}}_{a}}{\mathrm{dt}}=k_{a,\mathrm{TNF}}^{\mathrm{EC}}\mathrm{EC}C_{\mathrm{TNF}}+k_{a,\mathrm{IL}6}^{\mathrm{EC}}\mathrm{EC}C_{\mathrm{IL}6}+k_{a,\mathrm{IL}1}^{\mathrm{EC}}\mathrm{EC}C_{\mathrm{IL}1}-k_{\mathrm{da}}^{\mathrm{EC}}{\mathrm{EC}}_{a},$$ (4)

where *M* and $M_{a}$ denote resting and activated monocytes, respectively, and EC and ${\mathrm{EC}}_{a}$ denote resting and activated endothelial cells. $C_{\mathrm{TNF}}$, $C_{\mathrm{IL}6}$, and $C_{\mathrm{IL}1}$ represent TNF-α, IL-6, and IL-1𝛽 concentrations, respectively. These equations describe a phenomenological cytokine-to-cell activation relationship. The terms $k_{a,\mathrm{TNF}}^{M}$, $k_{a,\mathrm{IL}6}^{M}$, and $k_{a,\mathrm{IL}1}^{M}$ represent cytokine-dependent monocyte activation rates, whereas $k_{a,\mathrm{TNF}}^{\mathrm{EC}}$, $k_{a,\mathrm{IL}6}^{\mathrm{EC}}$, and $k_{a,\mathrm{IL}1}^{\mathrm{EC}}$ represent cytokine-dependent endothelial activation rates. The parameters $k_{\mathrm{da}}^{M}$ and $k_{\mathrm{da}}^{\mathrm{EC}}$ describe deactivation or loss of the activated cellular states.

Activated monocytes and endothelial cells were coupled to the coagulation module by increasing TF availability. TF dynamics were modeled as:

$$\begin{array}{l} \frac{d\mathrm{TF}}{\mathrm{dt}}=k_{t,\mathrm{TF}}+k_{t,\mathrm{TF}}^{M}M_{a}+k_{t,\mathrm{TF}}^{\mathrm{EC}}{\mathrm{EC}}_{a}-k_{d,\mathrm{TF}}\mathrm{TF}\\ \;-\left(k_{\mathrm{on},\mathrm{TF},\mathrm{VII}}\mathrm{TF}\cdot \mathrm{VII}-k_{\mathrm{off},\mathrm{TF},\mathrm{VII}}\mathrm{TF}:\mathrm{VII}\right)\\ \;-\left(k_{\mathrm{on},\mathrm{TF},\text{VIIa}}\mathrm{TF}\cdot \text{VIIa}-k_{\mathrm{off},\mathrm{TF},\text{VIIa}}\mathrm{TF}:\text{VIIa}\right). \end{array}$$ (5)

This equation provides the primary connection between inflammation and coagulation in the model. The basal term $k_{t,\mathrm{TF}}$ represents baseline TF production, whereas $k_{t,\mathrm{TF}}^{M}M_{a}$ and $k_{t,\mathrm{TF}}^{\mathrm{EC}}{\mathrm{EC}}_{a}$ represent TF up-regulation driven by activated monocytes and endothelial cells. TF is removed by first-order degradation and by binding to factor VII and factor VIIa to form TF:VII and TF:VIIa, respectively. Thus, cytokine-induced cellular activation increases TF availability, which promotes initiation of the extrinsic coagulation pathway.

The TF-initiated coagulation response was then propagated through formation of the active TF:VIIa complex and downstream TG. The coagulation module was built upon the mechanistic framework of Hockin et al. [36], including TF binding to factor VII/VIIa, activation of factors IX and X, formation of intrinsic tenase and prothrombinase complexes, TG from prothrombin, and inhibition by ATIII and TFPI. The TG profiles reported in the Hockin framework were used to verify that the reconstructed coagulation module preserved the expected TF-initiated coagulation behavior before it was coupled to the inflammation module. The core thrombin equation was:

$$\frac{d\mathrm{IIa}}{\mathrm{dt}}=k_{a,\mathrm{II}}^{\mathrm{Xa}}\mathrm{II}\cdot \mathrm{Xa}+k_{m,\mathrm{IIa}}^{\text{IIam}}\text{IIam}\cdot \mathrm{Va}:\mathrm{Xa}-k_{\mathrm{on},\text{ATIII},\mathrm{IIa}}\text{ATIII}\cdot \mathrm{IIa},$$ (6)

where II is prothrombin, IIa is thrombin, Xa is activated factor X, IIam is meizothrombin, and Va:Xa is the prothrombinase complex. The first 2 terms represent thrombin production through Xa-mediated prothrombin activation and prothrombinase-associated conversion of meizothrombin to thrombin. The final term represents thrombin inhibition through complex formation with ATIII. Following the Hockin–Mann coagulation model, TG was quantified using an activity-equivalent total thrombin signal that accounts for both thrombin and meizothrombin:

$${\mathrm{IIa}}_{\text{total}}\left(t\right)=\mathrm{IIa}\left(t\right)+1.2\;\text{IIam}\left(t\right),$$ (7)

where IIa denotes thrombin and IIam denotes meizothrombin. The coefficient 1.2 was adopted from the Hockin–Mann modeling convention to account for the higher thrombin-like activity of meizothrombin toward thrombin substrates [36]. This coefficient was not fitted in the present study and was not treated as an inflammation–coagulation coupling parameter. TG metrics, including lag time, TTP, peak thrombin, and endogenous thrombin potential (ETP), were calculated from ${\mathrm{IIa}}_{\text{total}}\left(t\right)$.

Endogenous anticoagulant feedback was represented through TFPI- and ATIII-mediated inhibition of activated coagulation complexes. TFPI dynamics were modeled as:

$$\begin{array}{c} \frac{d\text{TFPI}}{\mathrm{dt}}=k_{t,\text{TFPI}}-k_{d,\text{TFPI}}\left(\text{TFPI}+k_{d,\text{TFPI}}^{\mathrm{TNF}}C_{\mathrm{TNF}}\right)\\ \;-\left(k_{\mathrm{on},\mathrm{Xa},\text{TFPI}}\text{TFPI}\cdot \mathrm{Xa}-k_{\mathrm{off},\mathrm{Xa},\text{TFPI}}\text{TFPI}:\mathrm{Xa}\right)\\ \;-\left(k_{\mathrm{on},\text{TFPI},\text{TFVIIaXa}}\text{TFPI}\cdot \mathrm{TF}:\text{VIIa}:\mathrm{Xa}-k_{\mathrm{off},\text{TFPI},\text{TFVIIaXa}}\mathrm{TF}:\text{VIIa}:\mathrm{Xa}:\text{TFPI}\right). \end{array}$$ (8)

The corresponding TFPI inhibitory complexes were described as:

$$\begin{array}{c} \frac{d\left(\text{TFPI}:\mathrm{Xa}\right)}{\mathrm{dt}}=k_{\mathrm{on},\mathrm{Xa},\text{TFPI}}\text{TFPI}\cdot \mathrm{Xa}-k_{\mathrm{off},\mathrm{Xa},\text{TFPI}}\text{TFPI}:\mathrm{Xa}\\ -k_{\mathrm{on},\text{TFVIIa},\text{TFPIXa}}\mathrm{TF}:\text{VIIa}\cdot \text{TFPI}:\mathrm{Xa}, \end{array}$$ (9)

$$\begin{array}{c} \frac{d\left(\mathrm{TF}:\text{VIIa}:\mathrm{Xa}:\text{TFPI}\right)}{\mathrm{dt}}=k_{\mathrm{on},\text{TFVIIa},\text{TFPIXa}}\mathrm{TF}:\text{VIIa}\cdot \text{TFPI}:\mathrm{Xa}\\ +k_{\mathrm{on},\text{TFPI},\text{TFVIIaXa}}\text{TFPI}\cdot \mathrm{TF}:\text{VIIa}:\mathrm{Xa}-k_{\mathrm{off},\text{TFPI},\text{TFVIIaXa}}\mathrm{TF}:\text{VIIa}:\mathrm{Xa}:\text{TFPI}. \end{array}$$ (10)

These equations describe negative regulation of TF-initiated coagulation by TFPI. Free TFPI binds Xa to form TFPI:Xa, which can further inhibit TF:VIIa, thereby reducing propagation through the extrinsic pathway. The term $k_{d,\text{TFPI}}^{\mathrm{TNF}}C_{\mathrm{TNF}}$ represents TNF-*α*-associated reduction of effective TFPI availability, allowing inflammatory signaling to weaken this endogenous inhibitory pathway.

ATIII-mediated inhibition was modeled as:

$$\begin{array}{c} \frac{d\text{ATIII}}{\mathrm{dt}}=k_{t,\text{ATIII}}-k_{d,\text{ATIII}}\left(\text{ATIII}+k_{d,\text{ATIII}}^{\mathrm{IL}6}C_{\mathrm{IL}6}\right)-k_{\mathrm{on},\text{ATIII},\mathrm{IIa}}\text{ATIII}\cdot \mathrm{IIa}\\ \;-k_{\mathrm{on},\text{ATIII},\mathrm{Xa}}\text{ATIII}\cdot \mathrm{Xa}-k_{\mathrm{on},\text{ATIII},\mathrm{IXa}}\text{ATIII}\cdot \mathrm{IXa}-k_{\mathrm{on},\text{ATIII},\text{TFVIIa}}\text{ATIII}\cdot \mathrm{TF}:\text{VIIa}\\ \;-k_{\mathrm{on},\text{ATIII},\text{IIam}}\text{ATIII}\cdot \text{IIam}. \end{array}$$ (11)

The corresponding ATIII inhibitory complexes were:

$$\frac{d\left(\text{ATIII}:\mathrm{IIa}\right)}{\mathrm{dt}}=k_{\mathrm{on},\text{ATIII},\mathrm{IIa}}\text{ATIII}\cdot \mathrm{IIa},$$ (12)

$$\frac{d\left(\text{ATIII}:\mathrm{Xa}\right)}{\mathrm{dt}}=k_{\mathrm{on},\text{ATIII},\mathrm{Xa}}\text{ATIII}\cdot \mathrm{Xa},$$ (13)

$$\frac{d\left(\text{ATIII}:\mathrm{IXa}\right)}{\mathrm{dt}}=k_{\mathrm{on},\text{ATIII},\mathrm{IXa}}\text{ATIII}\cdot \mathrm{IXa},$$ (14)

$$\frac{d\left(\text{ATIII}:\mathrm{TF}:\text{VIIa}\right)}{\mathrm{dt}}=k_{\mathrm{on},\text{ATIII},\text{TFVIIa}}\text{ATIII}\cdot \mathrm{TF}:\text{VIIa},$$ (15)

$$\frac{d\left(\text{ATIII}:\text{IIam}\right)}{\mathrm{dt}}=k_{\mathrm{on},\text{ATIII},\text{IIam}}\text{ATIII}\cdot \text{IIam}.$$ (16)

These equations describe ATIII-dependent negative feedback on TG. ATIII inhibits thrombin, Xa, IXa, TF:VIIa, and meizothrombin through irreversible complex formation. The IL-6-associated term $k_{d,\text{ATIII}}^{\mathrm{IL}6}C_{\mathrm{IL}6}$ represents cytokine-mediated reduction of effective ATIII availability. Thus, the model captures 2 coupled inflammatory effects: cytokines increase the procoagulant drive by increasing TF availability and simultaneously reduce anticoagulant restraint by suppressing ATIII and TFPI activity.

Thrombin was also modeled as a feedback signal to the inflammatory subsystem. This feedback was motivated by experimental evidence linking thrombin signaling to inflammatory activation through PAR-1/NF-𝜅B-associated pathways [62,63]. Together, these ODEs define the core inflammation–coagulation coupling structure of the model. Cytokines activate monocytes and endothelial cells, activated cells increase TF production, TF initiates TG through the extrinsic pathway, and ATIII/TFPI provide endogenous inhibitory feedback. This formulation allows the model to quantify how inflammatory inputs shift TG dynamics through both procoagulant amplification and reduced anticoagulant regulation.

### Parameter sources and calibration strategy

Model parameters were assigned using a tiered strategy based on data availability and biological interpretation. Parameters in the TF-initiated coagulation module were primarily taken from the Hockin coagulation framework and related TG studies when direct kinetic values were available [36]. These parameters describe established biochemical reactions such as factor binding, enzymatic activation, complex formation, and inhibition by ATIII or TFPI. Parameters that could be derived from physiological constraints were calculated rather than fitted. For example, cytokine degradation rates were calculated from reported plasma half-lives as:

$$k_{\deg,i}=\frac{\ln\left(2\right)}{t_{1/2,i}},$$ (17)

where $t_{1/2,i}$ is the reported half-life of cytokine *i*. IL-1𝛽 and IL-6 were assigned plasma half-lives of approximately 1 h [57,58], whereas TNF-α was assigned a shorter half-life of approximately 4.6 min [59]. Baseline cytokine production rates were then determined by imposing a steady-state condition under the normal baseline state:

$$k_{\text{prod},i}=k_{\deg,i}C_{i,0},$$ (18)

where $C_{i,0}$ is the baseline concentration of cytokine *i*. This homeostatic calibration ensured that the inflammatory subsystem was physiologically grounded at baseline and that disease simulations represented deviations from normal inflammatory and hemostatic tone rather than artifacts of parameter initialization.

Parameters were fitted only when the corresponding reaction represented a phenomenological inflammation–coagulation coupling process for which direct kinetic constants were not available. These included parameters governing cytokine-dependent monocyte and endothelial cell activation, activated-cell-mediated TF production, TNF-α-associated TFPI suppression, IL-6-associated ATIII suppression, and thrombin-mediated cytokine amplification. The parameters associated with these coupling reactions were calibrated using published experimental datasets describing cytokine-dependent TF regulation, cytokine-associated anticoagulant suppression, and thrombin-related inflammatory responses [60–63].

For each calibration dataset, parameters were estimated using an iterative calibration strategy that combined objective-function-based optimization with manual refinement within physiologically plausible bounds. The optimization step minimized the weighted residual between experimental measurements and simulated outputs:

$$J\left(\theta \right)=\sum_{i=1}^{N}w_{i}{\left(y_{i}^{\exp}-y_{i}^{\mathrm{sim}}\left(\theta \right)\right)}^{2},$$ (19)

where θ denotes the fitted parameter set, $y_{i}^{\exp}$ represents the experimental or digitized experimental measurement, $y_{i}^{\mathrm{sim}}\left(\theta \right)$ is the corresponding model prediction, and $w_{i}$ is a weighting factor. When experimental data were reported as normalized fold changes, the corresponding model outputs were normalized to the same baseline before calibration. Following numerical optimization, selected parameters were manually refined when necessary to improve agreement with the experimental response magnitude, temporal trend, and steady-state behavior. All manual adjustments were restricted to biologically plausible ranges and were accepted only when they improved consistency between simulated and experimental data without producing negative concentrations or nonphysiological model behavior.

The coagulation module was first evaluated against TG profiles reported in the Hockin framework to verify that the reconstructed TF-initiated coagulation module reproduced the expected coagulation dynamics [36]. The inflammation–coagulation coupling parameters were then calibrated separately using experimental datasets describing cytokine-dependent TF up-regulation, ATIII or TFPI suppression, and thrombin-associated cytokine induction [60–63]. Finally, disease-specific virtual patient sampling ranges were calibrated using reported peak thrombin values. After calibration, the same sampling ranges were evaluated against lag time and TTP to validate whether the peak-calibrated virtual cohorts could also reproduce additional TG metrics. Therefore, agreement with peak thrombin was interpreted as calibration performance, whereas agreement with lag time and TTP was interpreted as a validation check of the calibrated sampling ranges.

Overall, the proposed mathematical model includes 38 reactions, 44 species, and 72 parameters. Details of the reaction network, species definitions, reaction rates, and parameter values are provided in the Supplementary Materials.

### Disease-specific virtual patient generation

Disease-specific virtual patient cohorts were generated to evaluate how inflammatory and coagulation factor variability shapes TG across distinct coagulation dysregulation states. Five cohorts were considered: normal subjects, COVID-19, SCD, T2DM, and hemophilia A. The disease groups were selected to span both inflammation-associated hypercoagulability and factor-deficiency-driven hypocoagulability.

For each disease condition, the sampling space was defined using literature-reported clinical measurements of coagulation factors, endogenous anticoagulants, and inflammatory cytokines. Therefore, the lower and upper bounds used for virtual patient generation were derived from clinically observed concentrations, activity ranges, or reported disease-specific variability, rather than arbitrary model-defined intervals. Sampled coagulation variables included prothrombin/factor II, factors V, VII, VIIa, VIII, IX, and X, as well as ATIII and TFPI. When disease-specific inflammatory data were available, IL-6, IL-1𝛽, and TNF-*α* were also included as sampled inflammatory inputs. All reported values were converted to consistent molar units before simulation. The corresponding ranges and references are summarized in Table 1.

**Table 1.** Range of coagulation and cytokine factors for normal subjects and subjects under 4 diseased conditions

Coagulation factors	COVID-19	T2DM	SCD
IL-6	232 (1–32,768) pg/ml [103]	4.1 (2.3–5.6) pg/ml [104]	60 ± 7 pg/ml [105]
TNF-α	25 (1–1,000) pg/ml [103]	3.8 (3.0–5.0) pg/ml [104]	122.3 ± 16.3 pg/ml [106]
IL-1𝛽	0.8 (0–32) pg/ml [103]	0.2 (0.2–0.3) pg/ml [104]	3.5 (0.0–27.26) pg/ml [107]
VII	0.5 mg/l (55%–170%) [108]	0.5 mg/l (60%–130%) [109]	0.31 mg/l (±7.5%) [110]
VIIa	0.2 mg/l (60%–130%) [109]	0.2165 mg/l ± 26.72% [111]	0.2 mg/l (60%–130%) [109]
IX	7.5 mg/l ± 50.6% [108]	5 mg/l (60%–130%) [109]	5 mg/l (60%–130%) [109]
X	11.1 mg/l ± 27.9% [108]	10 mg/l (60%–130%) [109]	6.6 mg/l ± 10% [110]
II	107.5 mg/l ± 31.2% [108]	100 mg/l (60%–130%) [109]	75 mg/l ± 10% [110]
VIII	0.262 mg/l ± 102.9% [108]	0.1 mg/l (50%–150%) [109]	0.2 mg/l (60%–130%) [109]
V	11.7 mg/l ± 44.9% [108]	10 mg/l (60%–130%) [109]	7.25 mg/l ± 17.5% [110]
TFPI	70 ng/ml [112]	197.56 ± 94.88 (pg/ml) [111]	70 ng/ml [112]
ATIII	0.134 mg/l ± 15.1% [108]	0.15 mg/l [113]	0.15 ng/ml [113]
Coagulation factors	Hemophilia	Normal	
IL-6	1–50 pg/ml [114]	<2.23 pg/ml [115]	
TNF-α	6.5–302.5 pg/ml [116]	<10.0 pg/ml [117]	
IL-1𝛽	153.6 ± 168.6 to 342.9 ± 134.8 pg/ml [118]	<1.34 pg/ml [115]	
VII	0.5 mg/l (60%–130%) [109]	10 mg/l (60%–130%) [109]	
VIIa	0.2 mg/l (60%–130%) [109]	0.5 mg/l (60%–130%) [109]	
IX	5 mg/l (60%–130%) [109]	5 mg/l (60%–130%) [109]	
X	10 mg/l (60%–130%) [109]	10 mg/l (60%–130%) [109]	
II	100 mg/l (60%–130%) [109]	100 mg/l (60%–130%) [109]	
VIII	<1% [119]	0.1 mg/l (60%–130%) [109]	
V	10 mg/l (60%–130%) [109]	10 mg/l (60%–130%) [109]	
TFPI	70 ng/ml [112]	70 ng/ml [112]	
ATIII	0.15 mg/l [113]	0.15 mg/l [113]	

ATIII, antithrombin III; SCD, sickle cell disease; T2DM, type 2 diabetes mellitus; IL-6, interleukin-6; IL-1𝛽, interleukin-1𝛽; TNF-α, tumor necrosis factor-α; TFPI, tissue factor pathway inhibitor

When clinical studies reported explicit lower and upper limits, those values were used directly as sampling bounds. When only summary statistics were available, such as mean, SD, or coefficient of variation, the sampling bounds were constructed from the reported central tendency and variability while enforcing non-negative and physiologically plausible values.

Latin hypercube sampling was used to generate 2,000 candidate virtual patients for each disease cohort within the disease-specific parameter ranges. For PRCC analysis, 5,000 Latin hypercube samples were generated for each disease group unless otherwise specified. This approach was used to efficiently sample the high-dimensional parameter space while maintaining broad coverage of interindividual variability across coagulation factors and inflammatory markers. Because patient-level covariance information among coagulation factors, anticoagulants, and cytokines was not consistently available across all disease groups, sampled variables were treated as independent during Latin hypercube sampling. This assumption was applied uniformly across cohorts and was considered when interpreting disease-specific virtual patient results.

After sampling, candidate parameter sets were screened for physiological and numerical plausibility. Samples were rejected post hoc if they produced negative concentrations, failed numerical integration, or generated physiologically implausible TG profiles, such as absent TG under conditions expected to support coagulation or extreme thrombin responses outside the clinically reported dynamic range.

After candidate virtual patients were generated, each parameter set was simulated under the corresponding disease-specific baseline condition. TG metrics were calculated from the simulated thrombin time course, including lag time, TTP, peak thrombin, and ETP. Based on the sensitivity analysis results, the sampling ranges of the most influential coagulation factors were adjusted using experimentally reported disease-specific peak thrombin values. Specifically, peak thrombin was used as the primary calibration target for defining TG-consistent virtual patient sampling ranges.

After calibration using peak thrombin, the calibrated sampling ranges were kept fixed and evaluated using lag time and TTP. These 2 metrics were not directly used to tune the sampling ranges and therefore served as additional validation metrics for assessing whether the calibrated virtual cohorts reproduced broader TG dynamics beyond the calibration target. The calibrated virtual cohorts were subsequently used to quantify the effects of inflammatory intensity and exposure duration on TG dynamics.

### Inflammatory exposure and repeated-cycle simulations

Inflammatory exposure simulations were performed to quantify how cytokine intensity and exposure duration alter TG. For each virtual patient cohort, baseline simulations were first performed without additional inflammatory scaling to establish the disease-specific TG profile. Pro-inflammatory exposure was then introduced by scaling IL-6, IL-1𝛽, and TNF-*α* relative to their baseline or disease-specific values. These scaled cytokine inputs were applied to the inflammation–coagulation coupling equations to modulate monocyte activation, endothelial cell activation, TF availability, ATIII suppression, and TFPI suppression.

For the cytokine-dose simulations, IL-6, IL-1𝛽, and TNF-α were scaled over predefined fold-change levels relative to their baseline or disease-specific values, including 0.5×, 1×, 2×, 5×, 10×, and 50× where applicable. The 5 pmol/l TF setting was used for the virtual patient baseline and inflammatory exposure simulations to provide a fixed coagulation trigger across cytokine conditions. In contrast, the separate single-cytokine calibration simulations used calibration-specific TF settings, including 2 pmol/l where applicable, according to the corresponding calibration datasets and simulation scripts. The baseline coagulation response was simulated in the absence of additional pro-inflammatory cytokine scaling and was used as the reference condition for evaluating cytokine-dependent changes in TG.

Because cytokine-mediated cellular activation and anticoagulant regulation occur on a longer time scale than TG, inflammatory exposure was simulated before initiating the coagulation phase. Specifically, the inflammation–coagulation coupling module was simulated for predefined exposure durations, including 12 and 24 h. The resulting cytokine-modulated cellular activation states, TF levels, ATIII levels, and TFPI levels were then used as initial conditions for the TG simulation. The coagulation phase was simulated over a seconds-to-minutes time scale, and the resulting thrombin time course was used to calculate lag time, TTP, peak thrombin, and ETP.

To provide an exploratory assessment of thrombin-mediated inflammatory feedback, repeated-cycle simulations were also performed as an auxiliary analysis. Each cycle consisted of an inflammatory exposure phase followed by a TG phase. The inflammatory exposure phase was simulated for 12 or 24 h, and the subsequent coagulation phase was simulated for 1,200 s. At the end of each coagulation phase, thrombin exposure from that cycle was used to update the inflammatory state in the next cycle when thrombin-mediated cytokine feedback was included. Coagulation factor concentrations were reset to their baseline values between cycles to isolate cytokine-mediated regulatory effects on subsequent TG responses. Because this reset assumption excludes cumulative coagulation factor consumption, the repeated-cycle analysis was treated as a simplified exploratory simulation rather than a physiological representation of progressive coagulopathy.

### Global sensitivity analysis and structural identifiability

Global sensitivity analysis was performed to quantify the influence of coagulation factors, endogenous anticoagulants, and inflammatory variables on TG metrics. Latin hypercube sampling was used to generate parameter sets across the predefined physiological or disease-specific ranges. For each sampled parameter set, the model was simulated and TG metrics were calculated, including lag time, TTP, peak thrombin, and ETP.

Before conducting parameter sensitivity analysis, we first evaluated the local structural identifiability of the coupled inflammation–coagulation model. The systems biology makeup language-based model was converted into an ODE system. For this structural identifiability test [64], each model state was specified as an observation function, representing an ideal full-state observation setting. Local structural identifiability was then assessed using a symbolic differential algebra-based workflow. This analysis was intended to determine whether the model structure permits unique local inference of states and parameters under ideal noise-free observations, rather than to evaluate practical identifiability under sparse or noisy experimental data. Because experimental calibration in this study was based primarily on TG outputs and separate cytokine- or factor-specific datasets rather than simultaneous observation of all model states, the structural identifiability result should not be interpreted as evidence of practical identifiability under the available experimental conditions. In practice, the experimentally available outputs are much more limited than this idealized setting. TG calibration primarily observes thrombin activity, whereas the inflammatory module is constrained using separate cytokine- or factor-specific datasets. Therefore, single-output or sparse-output observability may substantially reduce practical identifiability relative to the ideal full-state structural identifiability result.

PRCCs [65] were then calculated between each sampled parameter and each TG metric. PRCC analysis was selected because it quantifies monotonic relationships between uncertain model inputs and outputs while controlling for the effects of other sampled variables. A dummy variable with no mechanistic effect on the model was included as a negative control to estimate the background level of spurious correlation. Parameters with PRCC magnitudes greater than the dummy variable and statistically significant correlations were considered influential drivers of the corresponding TG metric.

Sensitivity analysis was performed in 2 stages. First, coagulation factor and anticoagulant levels were varied to identify the dominant drivers of baseline TG across disease-specific virtual cohorts. Second, all parameters were varied to evaluate how uncertainty in inflammation–coagulation coupling affected TG responses. These cytokine-related parameters included those governing monocyte activation, endothelial cell activation, activated-cell-mediated TF production, TNF-*α*-associated TFPI suppression, IL-6-associated ATIII suppression, and thrombin-mediated cytokine feedback.

### Numerical implementation and simulation settings

All simulations were implemented in MATLAB SimBiology Toolbox (MathWorks, Natick, MA). The ODE system was solved using the stiff solver ode15s, with both relative and absolute tolerances set to 10^−9^. Different simulation time windows were used depending on the biological process being simulated, because inflammatory regulation and TG occur on different time scales.

For TG simulations, the coagulation module was simulated on a seconds-to-minutes time scale, consistent with experimental TG assays, in which thrombin formation is typically measured over minutes. The simulated thrombin time course was used to calculate lag time, TTP, peak thrombin, and ETP. In contrast, experimental studies of cytokine-induced TF expression and cytokine-mediated regulation of anticoagulant pathways are commonly performed over an hours-long time scale [60,61]. Therefore, inflammatory exposure simulations were performed over longer durations, including 12 and 24 h, to allow cytokine-mediated monocyte activation, endothelial cell activation, TF up-regulation, ATIII suppression, and TFPI suppression before initiating the TG phase. For repeated-cycle simulations, each cycle consisted of an inflammatory exposure phase followed by a TG phase, reflecting the distinct time scales of inflammatory regulation and coagulation activation.

All reported cytokine, coagulation factor, and inhibitor concentrations were converted to consistent molar units before simulation. Thus, cytokine variables in the ODE system represent dimensional molar concentrations rather than dimensionless normalized quantities. When fold-change perturbations were applied, the fold changes were imposed as multiplicative scalings of the corresponding baseline or disease-specific molar cytokine concentrations. Normalization was used only for comparison with experimental datasets that reported relative fold-change measurements, not for defining the internal cytokine state variables. Simulation outputs were converted to the units and time scales used in the corresponding experimental datasets for comparison. TG curves were reported in seconds or minutes, whereas inflammatory exposure durations were reported in hours. ImageJ software (National Institutes of Health) [66] and GetData Graph Digitizer were used for quantitative digitization of published Western blot and other experimental datasets when numerical values were not directly available.

### Clinical data for creating virtual patients

We generated virtual patient cohorts to investigate how the extent and duration of inflammation alter TG dynamics across 4 distinct diseases: COVID-19-associated coagulopathy, SCD, T2DM, and hemophilia A. Clinical laboratory ranges for coagulation factors and inflammatory markers were collected for each disease group, as summarized in Table 1. To construct these cohorts, we employed Latin hypercube sampling to efficiently explore parameter spaces and generate physiologically diverse virtual patients. Using disease-specific distributions (mean and SD) of coagulation factors and cytokines as reference values, we generated 2,000 virtual patients per condition. Peak thrombin was used as the primary calibration target, whereas lag time and TTP were used as secondary evaluation metrics to assess whether the calibrated virtual cohorts reproduced broader TG dynamics [67–70]. This calibration step ensured that the simulated patients exhibited physiologically consistent coagulation profiles aligned with observed TG dynamics. Finally, using the TG curve as a quantitative biomarker, we systematically analyzed how pro-inflammatory cytokines modulate TG dynamics across the 4 disease states, thereby elucidating disease-specific thrombo-inflammatory mechanisms.

## Results

### Calibration of inflammation–coagulation coupling parameters and validation of the coagulation module

**Fig. 2.**
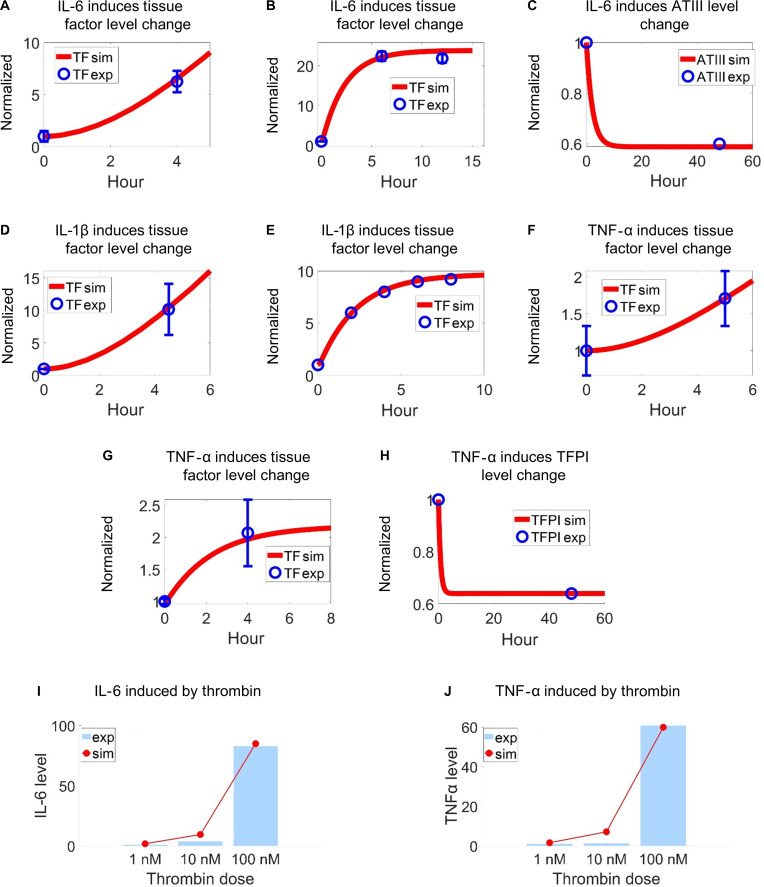
Calibration of inflammation–coagulation coupling mechanisms. (A to C) interleukin-6 (IL-6)-induced tissue factor (TF) up-regulation and antithrombin III (ATIII) suppression over time [71–73]. (D and E) Interleukin-1𝛽 (IL-1𝛽)-induced TF expression dynamics [74,75]. (F to H) Tumor necrosis factor-α (TNF-α)-induced TF up-regulation and tissue factor pathway inhibitor (TFPI) suppression [76–78]. (I and J) Thrombin-induced cytokine production, showing dose-dependent induction of IL-6 and TNF-α [79]. Model simulations are shown as red lines and experimental measurements as blue markers. Outputs were normalized to baseline levels where appropriate.

We first calibrated the inflammation–coagulation coupling parameters using experimental datasets that characterize cytokine-mediated regulation of coagulation-related factors and thrombin-mediated inflammatory feedback. Specifically, cell-type-specific data were used to constrain the effects of IL-6, IL-1𝛽, and TNF-α on TF expression in monocytes and endothelial cells. As shown in Fig. 2A to C, IL-6-induced TF up-regulation and ATIII suppression were calibrated using reported experimental measurements [71–73]. IL-1𝛽-induced TF expression dynamics were calibrated using the datasets shown in Fig. 2D and E [74,75], whereas TNF-α-induced TF up-regulation and TFPI suppression were calibrated using the data summarized in Fig. 2F to H [76–78]. These cytokine-mediated regulatory mechanisms were included to represent the procoagulant shift associated with elevated inflammatory burden. In addition, thrombin-induced cytokine production was calibrated using experimental data obtained under different thrombin concentrations, as shown in Fig. 2I and J [79]. Together, these calibration steps constrained the bidirectional coupling between inflammatory signaling and coagulation activation.

**Fig. 3.**
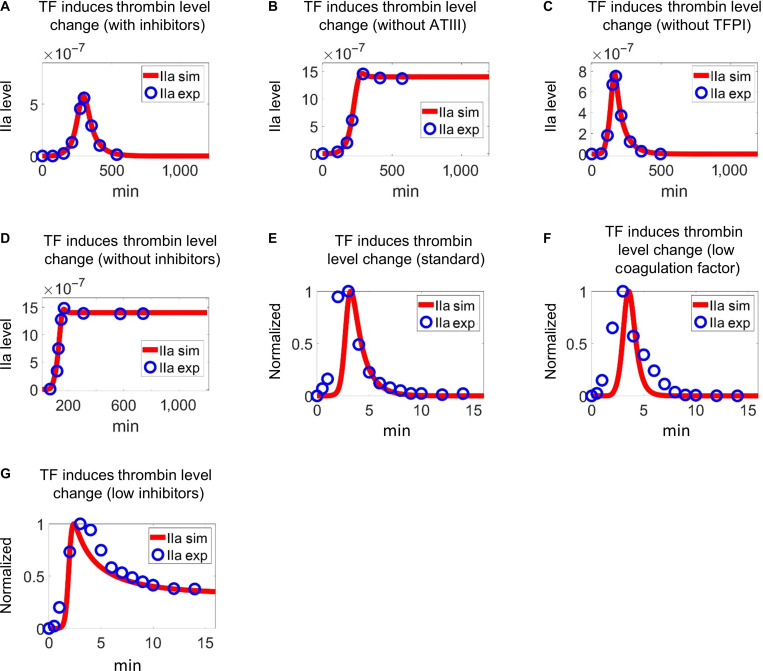
Validation of the coagulation module using tissue factor (TF)-driven thrombin generation (TG) profiles under different inhibitory and plasma protein conditions. The coagulation module was first benchmarked against published TF-induced TG simulations from the reference coagulation model. (A to D) TG responses in the presence of both antithrombin III (ATIII) and tissue factor pathway inhibitor (TFPI) (A), in the absence of ATIII (B), in the absence of TFPI (C), and in the absence of both ATIII and TFPI (D). Reference simulation data used in (A) to (D) were adopted from [36]. The coagulation module was further evaluated using independent experimental TG data. (E to G) TF/FVIIa-initiated TG under standard mean plasma protein concentrations (E), under a procoagulant-favored condition in which prothrombin and factors V, VIII, IX, and X were set to 150% and the anticoagulants ATIII and TFPI were set to 50% of their mean plasma concentrations (G), and under an anticoagulant-favored condition in which procoagulants were set to 50% and anticoagulants were set to 150% of their mean plasma concentrations (F). Experimental data used in (E) to (G) were adopted from [80]. Together, these comparisons support the ability of the coagulation module to reproduce TF-driven TG dynamics across standard plasma, inhibitor-deficient, procoagulant-favored, and anticoagulant-favored conditions.

After calibrating the inflammation-related coupling parameters, we evaluated the coagulation module using TF-driven TG profiles under multiple inhibitory and plasma protein perturbation conditions. The coagulation module was first benchmarked against published simulations from a reference coagulation model under the same 25 pM TF stimulation [36]. Figure 3A to D compares thrombin dynamics simulated by the present coagulation module with those generated by the reference model. Specifically, TG responses were evaluated in the presence of both ATIII and TFPI (Fig. 3A), in the absence of ATIII (Fig. 3B), in the absence of TFPI (Fig. 3C), and in the absence of both ATIII and TFPI (Fig. 3D). Across these conditions, the model reproduced the major dynamical features of the reference simulations, including thrombin initiation timing, peak amplitude, and sustained or decaying response profiles. The consistency of the responses under anticoagulant perturbations indicates that the coagulation module preserves the key regulatory effects of ATIII and TFPI represented in the reference framework.

To further validate the physiological behavior of the coagulation module, we simulated TG dynamics under independent experimental conditions reported for normal plasma [80]. TG was initiated with 5 pM factor VIIa-TF in the presence of all coagulation proteins. Model predictions were compared against 3 experimental scenarios: baseline plasma protein concentrations (Fig. 3E); a procoagulant-favored condition in which prothrombin and factors V, VIII, IX, and X were set to 150% while ATIII and TFPI were set to 50% of their mean plasma concentrations (Fig. 3G); and an anticoagulant-favored condition in which procoagulant factors were set to 50% while ATIII and TFPI were set to 150% of their mean plasma concentrations (Fig. 3F). Under baseline conditions, the model reproduced the characteristic thrombin burst followed by decay. In the procoagulant-favored condition, reduced inhibitory capacity and elevated procoagulant factor levels produced enhanced and more sustained thrombin activity. Conversely, in the anticoagulant-favored condition, increased inhibitory capacity resulted in attenuated TG. Overall, these comparisons support the ability of the coagulation module to reproduce TF-driven TG dynamics across standard plasma, inhibitor-deficient, procoagulant-favored, and anticoagulant-favored conditions.

After validating the calibrated model against both reference simulations and experimental TG data, we further assessed whether the model structure itself was locally identifiable. Local structural identifiability analysis was performed on the systems biology makeup language-derived ODE system, which included 41 dynamic state variables. Under an ideal full-state observation setting, where each state was defined as an observation function, $y_{i}\left(t\right)=x_{i}\left(t\right)$ for i=1,…,41, all state variables and all tested kinetic parameters were locally structurally identifiable (Table S1). This result supports the structural well-posedness of the coupled inflammation–coagulation model and indicates that the calibrated model does not exhibit intrinsic local structural nonidentifiability under the specified observation setting. Thus, any remaining uncertainty in model calibration or virtual patient generation is more likely due to practical factors, including limited experimental observables, measurement noise, and parameter correlations.

### Inflammatory priming drives nonlinear thrombin amplification and a self-reinforcing thromboinflammatory loop

To evaluate how inflammatory priming alters coagulation output, we analyzed both ATIII−IIa complex formation and active TG. ATIII−IIa was used as an indicator of cumulative thrombin neutralization, whereas the TG curve was used to characterize the temporal kinetics of coagulation activation under cytokine-mediated perturbation.

**Fig. 4.**
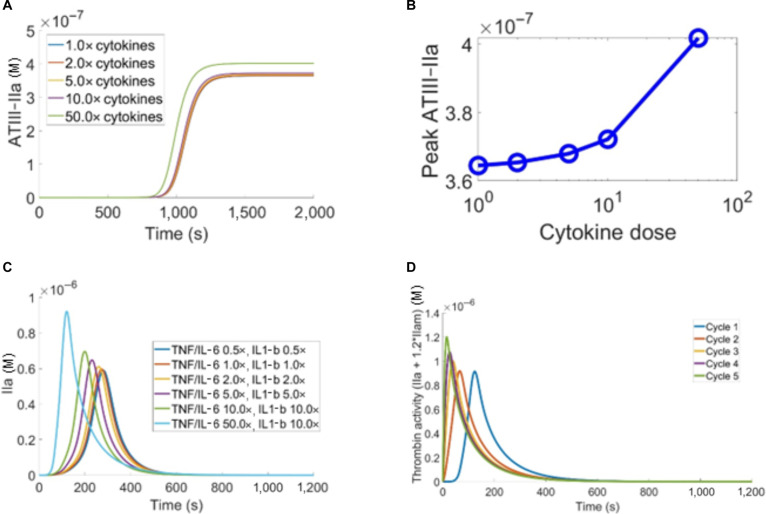
Simulated effects of pro-inflammatory cytokines on coagulation dynamics. (A) The dynamics of antithrombin III-IIa (ATIII-IIa) generation by different levels of cytokines. (B) The total amount of ATIII-IIa generated by different levels of cytokines. (C) The thrombin generation (TG) dynamics at different levels of cytokines. In (A) to (C), cytokine concentrations were represented as dimensional molar quantities, and fold-change labels indicate multiplicative scaling relative to the normal baseline cytokine concentrations. (D) Auxiliary repeated-cycle simulation under the reset-factor assumption. This panel illustrates exploratory feedback-dependent changes in thrombin activity and does not represent cumulative coagulation factor consumption.

Figure 4A shows that increasing cytokine stimulation produced a dose-dependent increase in ATIII−IIa complex accumulation while preserving the overall sigmoidal shape of the response. Across cytokine conditions, low cytokine scaling resulted in nearly overlapping profiles with similar lag phases. Notably, higher inflammatory stimulation (e.g., 50.0× cytokines) not only induced an evident upward shift in the terminal ATIII−IIa level but also caused an earlier onset of accumulation, indicating accelerated neutralization kinetics under severe inflammation.

Quantification of this accumulation further revealed a nonlinear dependence on cytokine intensity (Fig. 4B). The peak ATIII−IIa level changed only modestly across low-to-intermediate cytokine stimulation but increased more prominently once the inflammatory input exceeded a higher-intensity range. This pattern suggests that the inflammation–coagulation coupling response exhibits stronger procoagulant amplification primarily under severe inflammatory challenges, whereas it remains relatively insensitive to lower-grade cytokine perturbations.

To further resolve the temporal dynamics of this amplification, we examined the activity-equivalent total TG profiles (Fig. 4C). Consistent with the cumulative trends observed in ATIII−IIa, scaling up cytokine doses led to a pronounced, dose-dependent elevation of the peak thrombin concentration alongside a progressive left shift in the TTP, highlighting an accelerated and amplified procoagulant response.

While the single-hit scaling experiments (Fig. 4A to C) characterize the unidirectional impact of inflammatory priming on coagulation, TG from coagulation conversely exerts a positive feedback effect on inflammatory mediator production via PAR−1/NF−κB signaling [62,63], perpetuating a self-sustaining inflammation–thrombosis cycle. To further examine the feedback behavior between inflammation and coagulation, we analyzed TG across repeated inflammation–coagulation cycles. This analysis was used to determine whether prior coagulation activation could reshape subsequent thrombin responses under sustained inflammatory stimulation. Compared with a single inflammatory perturbation, the repeated-cycle simulation allowed evaluation of history-dependent changes in thrombin activity and provided insight into how persistent inflammatory signaling may alter the coagulation response over successive rounds of activation.

As an auxiliary analysis, Fig. 4D shows thrombin activity across repeated inflammation–coagulation cycles under the reset-factor assumption. In this simplified setting, thrombin activity increased across cycles, suggesting that thrombin-mediated inflammatory feedback may influence subsequent TG responses through cytokine-associated TF up-regulation and attenuation of endogenous anticoagulant regulation. This behavior is broadly consistent with the established concept that persistent inflammatory signaling and coagulation activation can reinforce one another in thromboinflammatory disorders, including severe COVID-19 and sepsis [11,26,81–83]. However, because coagulation factor concentrations were reset to baseline between cycles, this result should be interpreted only as an exploratory indication of feedback-driven history dependence, not as a direct simulation of cumulative coagulation factor depletion, microvascular thrombosis, organ dysfunction, or progressive coagulopathy.

### Enhancing TG assay simulations across virtual patient cohorts

In this section, we used the proposed model to evaluate TG dynamics across virtual patient cohorts representing 4 distinct disease states: COVID-19 coagulopathy, SCD, T2DM, and hemophilia A. We first applied the disease-specific coagulation factor ranges listed in Table 1 to the coagulation module and compared commonly used TG metrics derived from the simulated TG curves, including lag time, TTP, peak thrombin concentration, and ETP, against experimentally reported values for COVID-19 coagulopathy [67], SCD [69], T2DM [68], and hemophilia A [70]. The initial uncalibrated virtual patient sampling ranges did not consistently reproduce the experimental TG metrics across the 4 disease conditions, indicating that disease-specific calibration of the virtual patient sampling ranges was necessary before the model could quantitatively reproduce patient-level TG dynamics. This precalibration discrepancy is summarized in Figs. S5 to S7, which compare uncalibrated simulations, calibrated simulations, and experimental measurements for peak thrombin, TTP, and lag time, respectively.

**Fig. 5.**
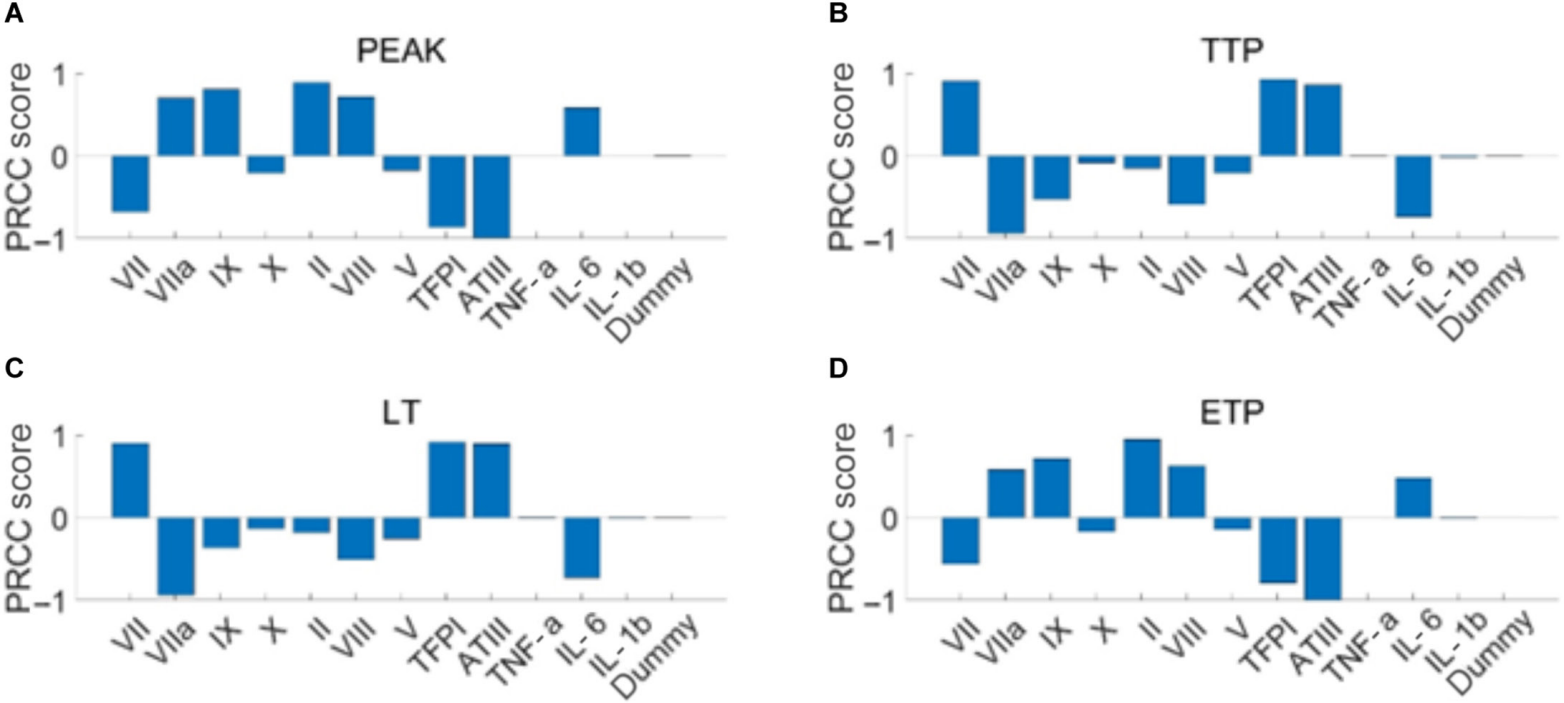
Sensitivity analysis of inflammatory and coagulation factors on thrombin generation (TG) dynamics. Global sensitivity analysis using partial rank correlation coefficients (PRCCs) quantified the influence of coagulation factors, anticoagulants, and inflammatory mediators on TG metrics, including peak thrombin (A), time to peak (TTP) (B), lag time (LT) (C), and endogenous thrombin potential (ETP) (D). The dummy variable acts as a control, representing a parameter with zero influence on the model output.

To calibrate the model effectively and efficiently, we first performed sensitivity analysis using Latin hypercube sampling and PRCC analysis, following the algorithm described by Marino et al. [65]. The analysis targeted 4 TG metrics: peak thrombin concentration, TTP, lag time, and ETP. For each disease group, 5,000 simulations were performed. The relative influence of each coagulation and inflammatory factor on these metrics for COVID-19 virtual patients is summarized in Fig. 5A to D. In addition, the complete parameter-level PRCC analysis from the 2-stage inflammation–coagulation simulation workflow is provided in Figs. S1 to S4. These supplementary analyses summarize the monotonic associations between sampled model parameters and ETP, lag time, peak thrombin, and TTP, respectively, with parameters ordered by absolute PRCC magnitude.

Our results show that factor II concentration exerted the strongest positive effect on peak thrombin levels (Fig. 5A) and ETP (Fig. 5D), whereas ATIII showed the strongest negative association with both end points. Inflammatory mediators also displayed positive effects on peak thrombin and ETP, with IL-6 exhibiting a more pronounced influence than the other inflammatory factors considered. Notably, although factor VII is generally expected to support TF-dependent coagulation initiation, PRCC analysis revealed a negative association between factor VII and both peak thrombin and ETP in the present model setting. This result should be interpreted as a model-dependent emergent behavior rather than as direct experimental evidence that increased factor VII suppresses TG in vivo. One possible explanation is that, under fixed TF availability and the Hockin-derived reaction structure, increased factor VII may compete with factor VIIa for TF binding, thereby reducing the effective availability of catalytically active TF–VIIa complexes. Therefore, this negative association should be viewed as conditional on the current model formulation and simulation settings. Consistent trends were observed for the temporal TG metrics. Procoagulant and inflammatory factors generally shortened TTP (Fig. 5B) and lag time (Fig. 5C), corresponding to accelerated TG, with factor VIIa showing one of the strongest time-shortening effects.

Based on the sensitivity analysis and the precalibration comparison, peak thrombin was selected as the primary calibration target for disease-specific virtual patient generation. Specifically, the disease-specific sampling ranges of coagulation factors were adjusted so that the simulated peak thrombin distributions aligned with experimentally measured peak thrombin values reported for hemophilia A, COVID-19, SCD, and T2DM [67–70], while remaining within physiologically and clinically relevant bounds. Figure S5 illustrates the precalibration discrepancy in peak thrombin and provides the rationale for this peak-based tuning step.

**Fig. 6.**
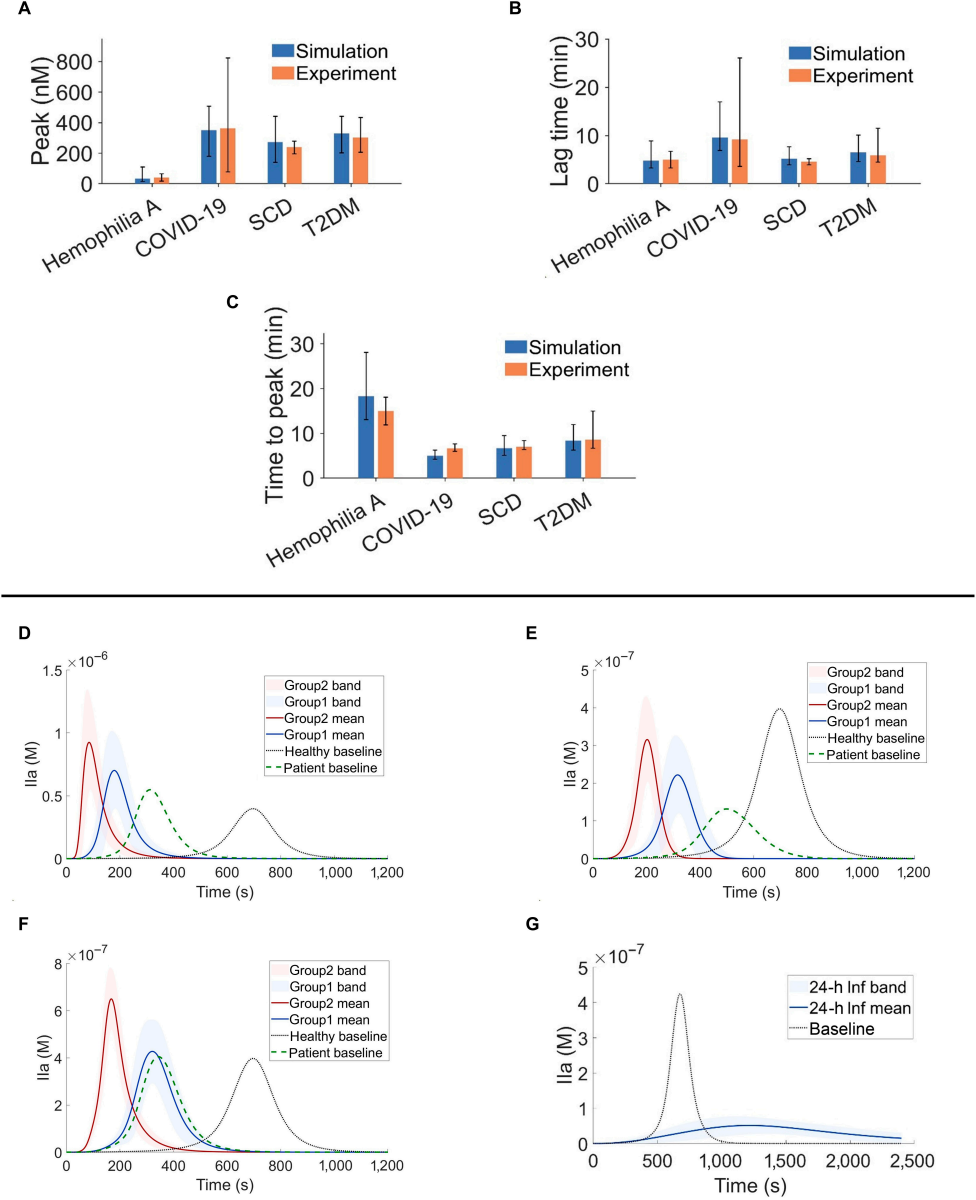
Thrombin generation (TG) dynamics of virtual patients under 4 disease conditions. Comparison of simulated and experimental TG metrics, including peak (A), lag time (B), and time to peak (C), across hemophilia A, COVID-19, sickle cell disease (SCD), and type 2 diabetes mellitus (T2DM). A total of 2,000 virtual patients are generated for each disease condition. TG profiles of virtual patients of COVID-19 (D), SCD (E), and T2DM (F) under baseline conditions and inflammatory exposure (12 h [group1] and 24 h [group2]), shown as mean trajectories with variability bands. (G) TG profiles of virtual patients of hemophilia A under baseline conditions. Virtual patient ensembles were constructed by sampling coagulation and inflammatory parameters within disease-specific physiological ranges.

After calibration using peak thrombin (Fig. 6A), the calibrated sampling ranges were kept fixed and further evaluated using 2 additional TG metrics, lag time and TTP, shown in Fig. 6B and C, respectively. Because lag time and TTP were not directly used to tune the sampling ranges, their agreement with the corresponding experimental measurements provided an independent validation check of the calibrated virtual patient generation strategy. This validation logic is further supported by Figs. S6 and S7, which show the corresponding precalibration comparisons for TTP and lag time. Overall, the simulated TG metrics generally fell within the experimental variability ranges across the 4 examined patient groups, indicating that the proposed virtual patient pipeline can quantitatively reproduce disease-specific TG dynamics. These calibrated virtual patient populations were then used as baseline conditions for downstream analyses of the impact of pro-inflammatory cytokines.

Next, we examined the impact of pro-inflammatory cytokines on TG curves for virtual patient groups representing COVID-19 (Fig. 6D), SCD (Fig. 6E), and T2DM (Fig. 6F). Pro-inflammatory cytokine exposure was simulated at 2 durations, 12 and 24 h, to assess how exposure duration modulates coagulation response.

For COVID-19 (Fig. 6D), both 12- and 24-h inflammatory exposures produced a markedly elevated thrombin peak relative to the uninflamed baseline and normal subjects, accompanied by shortened lag time and earlier TTP. This pattern is consistent with the hypercoagulable state associated with COVID-19 coagulopathy and suggests that cytokine-mediated inflammatory priming can enhance and accelerate TG. The 24-h exposure further increased TG compared with the 12-h exposure, indicating a duration-dependent inflammatory effect.

In SCD (Fig. 6E), inflammatory exposure also shortened lag time and advanced peak formation. However, the peak thrombin magnitude remained lower than that observed in normal subjects. This apparent constraint may result from disease-specific alterations in the coagulation factor profile, including reduced or consumed plasma factor availability under chronic coagulation activation. In particular, ongoing low-grade coagulation activity in SCD may reduce the availability of downstream coagulation factors, including factor X, thereby constraining the maximum thrombin burst even under inflammatory stimulation [4,84–86].

For T2DM (Fig. 6F), the TG curves qualitatively mirrored those of COVID-19, exhibiting shortened lag time, earlier peak formation, and elevated thrombin peak relative to the uninflamed baseline and normal subjects. These features collectively indicate inflammation-driven enhancement of coagulation activity. Across COVID-19, SCD, and T2DM virtual cohorts, prolonged inflammatory stimulation generally produced earlier thrombin onset and higher peak IIa levels than shorter exposure, supporting a duration-dependent effect of inflammatory priming on TG dynamics.

In contrast to these inflammation-associated hypercoagulable conditions, the hemophilia A virtual patient population exhibited a distinct response pattern (Fig. 6G). Under baseline conditions without additional inflammatory signaling, TG remained markedly attenuated, consistent with a hypocoagulable phenotype. This constrained thrombin amplification reflects the dominant effect imposed by factor VIII deficiency. Together, these results demonstrate that TG-calibrated virtual patient populations can capture both inflammation-driven hypercoagulability and disease-specific constraints on TG.

## Discussion and Summary

In this study, we developed and calibrated a mechanistic inflammation–coagulation model and employed it in a virtual patient framework to investigate how inflammatory burden reshapes coagulation under hyperinflammatory conditions [11,12,23]. The use of an ODE-based framework is consistent with prior computational coagulation models in which coagulation reactions can be represented using first- and second-order kinetics, together with enzyme-mediated saturation mechanisms [31,36]. The model encodes cytokine-mediated up-regulation of TF in monocytes and endothelial cells, concurrent suppression of endogenous anticoagulants, specifically ATIII and TFPI, and positive feedback from thrombin to inflammatory signaling [15,22,87–89], which enables quantification of how the increasing inflammatory intensity and duration systematically alters TG at different disease conditions [3,4,15,90]. Our results show that the virtual patient simulations qualitatively reproduce trends consistent with clinical observations across different hypercoagulable diseases, including COVID-19, SCD, and T2DM, demonstrating the feasibility of the model in simulating thrombo-inflammatory scenarios [3,4,90,91]. Furthermore, our model also provides an exploratory indication that thrombin-mediated inflammatory feedback may influence subsequent TG responses under simplified reset-cycle conditions [22,23]. This systems-level perspective provides a mechanistic explanation for why inflammatory states are frequently accompanied by heightened coagulation activity and thrombotic risk in severe inflammatory contexts [92,93].

The inflammation intensity analysis in Fig. 4A to C highlights the nonlinear nature of inflammation–coagulation coupling. The increasing inflammatory burden leads to a nonlinear increase in TG, suggesting that coagulation activation is disproportionately sensitive to high cytokine levels. Importantly, virtual patient simulations demonstrate that both inflammatory intensity and duration critically impact the coagulation dynamics. Prolonged inflammatory stimulation resulted in earlier thrombin initiation and higher peak levels in COVID and T2DM patients compared with those with shorter exposure. These findings emphasize the importance of cumulative inflammatory burden rather than transient cytokine elevations, aligning with clinical observations linking sustained inflammation, endothelial dysfunction, cytokine persistence, and coagulation activation to progressive hypercoagulability and thrombotic risk [3,92,94–96].

Disease-specific simulations revealed both shared and divergent TG dynamics under inflammatory conditions. In inflammatory diseases such as COVID-19 and T2DM, inflammatory signaling markedly amplified TG and accelerated coagulation initiation, consistent with clinical and experimental evidence of hypercoagulability in these settings [92,97–99]. These findings are also consistent with recent computational studies suggesting that diabetes-associated changes in inflammatory state, blood properties, and coagulation-related variables may increase clot formation risk [5,6]. In contrast, the hemophilia A virtual population exhibited fundamentally attenuated TG dynamics, driven by factor VIII deficiency that constrains TG irrespective of inflammatory stimulus. This divergent behavior underscores the model’s capacity to capture a key mechanistic distinction between diseases in which inflammation acts as the primary driver of coagulation dysregulation and those in which an intrinsic factor deficiency suppresses the system-level coagulation activity.

Several limitations should be acknowledged and considered in the future work. First, the model uses TG as a surrogate marker of coagulation activation and does not explicitly describe downstream fibrinogen-to-fibrin conversion, fibrin clot formation, clot propagation, or fibrinolysis. Therefore, it cannot capture the complete progression from coagulation initiation to thrombus formation and clot resolution. This is particularly relevant under inflammatory conditions, where fibrinogen is itself inflammation-associated and has been reported to increase in COVID-19 patients with excessive inflammation and severe disease [19]. Future extensions could incorporate inflammation-dependent fibrinogen regulation and couple the current TG module with fibrin formation, fibrinolysis, or flow-dependent coagulation models [53]. Mechanistically, elevated fibrinogen may increase fibrin formation downstream of thrombin, alter fibrin network density and clot stiffness, and contribute to resistance to fibrinolysis. Fibrin and fibrinogen may also interact with platelets, leukocytes, and endothelial cells, thereby linking clot formation to inflammatory feedback pathways. Incorporating these processes would allow future models to connect cytokine-enhanced TG with clot structure, clot persistence, and thrombus resolution. Relatedly, the current formulation is a well-mixed ODE system and does not include spatial transport, blood flow, shear stress, endothelial surface heterogeneity, or thrombus growth under flow, although these factors can strongly affect local coagulation and thrombosis risk. Computational fluid dynamics-based and digital twin studies have emphasized the importance of integrating coagulation kinetics with patient-specific flow environments, especially in vascular and cardiac thrombosis modeling [52–54].

Second, the inflammatory module is intentionally reduced and phenomenological. It represents selected cytokine-mediated procoagulant effects through IL-6, IL-1𝛽, TNF-*α*, monocyte/endothelial activation, TF up-regulation, and ATIII/TFPI suppression, but it should not be interpreted as a comprehensive model of systemic inflammatory coagulopathy. Important nonmodeled mechanisms include IL-10-mediated anti-inflammatory resolution, IL-12, IFN-𝛾, NETosis, complement activation, platelet immune signaling, endothelial glycocalyx disruption, protein C pathway regulation, and fibrinolytic feedback [11,23,100–102]. As a result, the model may underestimate or misrepresent inflammation–coagulation coupling when these mechanisms dominate.

Third, although local structural identifiability analysis indicates that the model is structurally identifiable under idealized full-state observation, this does not guarantee practical identifiability under the available experimental conditions. The model includes many kinetic and phenomenological coupling parameters, whereas calibration relies on TG profiles and separate cytokine- or factor-specific datasets. Parameter correlations and nonunique parameter combinations may therefore persist. Accordingly, the PRCC analysis should be interpreted as identifying influential parameters for TG metrics, not as resolving practical identifiability or eliminating parameter uncertainty.

Finally, the virtual patient framework captures variability in selected coagulation and inflammatory factors but has not yet been directly calibrated or prospectively validated using individual patient-level longitudinal data due to the limited data availability. In addition, TG data used for virtual patient calibration were obtained from different laboratories and protocols, which may introduce interlaboratory variability and limit direct cross-disease comparison. Recent patient-specific coagulation modeling and hybrid neural-network–ODE studies suggest possible routes for linking clinical variables, such as white blood cell count, D-dimer, disease status, or residual thrombotic burden, to mechanistic parameters [55,56].

In summary, this study introduces a mechanistic, TG-calibrated virtual patient framework that elucidates how inflammatory burden shapes coagulation dynamics. By incorporating interindividual variability, the proposed computational approach extends conventional TG assays to account for the interplay between inflammation and coagulation. While further validation with patient-level longitudinal data is required, this framework provides a foundation for mechanistically interpreting thromboinflammatory progression and generating disease-specific hypotheses.

## Data Availability

All data and code needed to evaluate the conclusions of the study are available in the Supplementary Materials and deposited at https://github.com/dpdclub/Computational-modeling-of-pro-inflammatory-cytokine-enhanced-blood-coagulation/blob/main/code_new.zip.
