# Supplementary material for: Computational Modeling of Pro-inflammatory Cytokine-Enhanced Blood Coagulation
Source: Comput Struct Biotechnol J. 2026 Aug 5;35(1):0180. doi: 10.34133/csbj.0180 (PMC13438037; doi:10.34133/csbj.0180)
Supplement: Supplementary file 1 [file csbj.0180.f1.zip › supplement_version2.docx]

**Supplementary Information**

**Structural identifiability, parameter sensitivity analysis, and pre-calibration comparison of the inflammation--coagulation model**

This combined supplement presents the local structural identifiability results first, followed by the parameter sensitivity analysis figures and the pre-calibration comparison figures. The original calibrated Supplementary Figure S1 from the calibration file was removed before merging. The PRCC figures summarize parameter sensitivity for thrombin generation outputs in the two-stage coagulation-inflammation workflow. The remaining pre-calibration figures summarize the uncalibrated virtual patient sampling ranges relative to experimental and calibrated simulation metrics.

**Supplementary Methods Summary**

For the local structural identifiability analysis, the SBML-derived model was exported to a ordinary differential equation system, with each dynamic state included as an observable output in the tested observation setting. Structural local identifiability was assessed using the exported ODE and observation equations. For the parameter sensitivity analysis, a two-stage simulation workflow was used. Phase 1 simulated inflammatory activation with coagulation factors turned off. Phase 2 introduced fixed nominal coagulation factor initial amounts and simulated thrombin generation. Latin hypercube sampling was applied to model parameters around their nominal values, whereas species initial conditions were not sampled. Thrombin generation outputs were summarized using peak thrombin generation (PEAK), time to peak thrombin (TTP), endogenous thrombin potential (ETP), and lag time (LT). Partial rank correlation coefficients (PRCCs) were calculated to quantify the monotonic association between each sampled parameter and each thrombin generation metric. For the virtual patient calibration workflow, disease-specific sampling ranges were tuned using experimental peak thrombin values. Lag time and time to peak were then evaluated as validation metrics.

**Supplementary Table S1.**

Local structural identifiability results for the coupled inflammation--coagulation model. The table lists all dynamic state variables and all kinetic parameters returned as locally identifiable in the current local structural identifiability assessment.

| **Category** | **Quantity** | **Model name / description** | **Local structural identifiability** |
| --- | --- | --- | --- |
| Parameter | ka10_9a | Kinetic model parameter | Locally identifiable |
| Parameter | ka1_b2a | Kinetic model parameter | Locally identifiable |
| Parameter | ka2_5a10a | Kinetic model parameter | Locally identifiable |
| Parameter | ka2_b10a | Kinetic model parameter | Locally identifiable |
| Parameter | ka5_b2a | Kinetic model parameter | Locally identifiable |
| Parameter | ka7_b10a | Kinetic model parameter | Locally identifiable |
| Parameter | ka7_b2a | Kinetic model parameter | Locally identifiable |
| Parameter | ka7_b7ac | Kinetic model parameter | Locally identifiable |
| Parameter | ka8_b2a | Kinetic model parameter | Locally identifiable |
| Parameter | ka9_tf7a | Kinetic model parameter | Locally identifiable |
| Parameter | ka_il12e | Kinetic model parameter | Locally identifiable |
| Parameter | ka_il12m | Kinetic model parameter | Locally identifiable |
| Parameter | ka_il62e | Kinetic model parameter | Locally identifiable |
| Parameter | ka_il62m | Kinetic model parameter | Locally identifiable |
| Parameter | ka_t2e | Kinetic model parameter | Locally identifiable |
| Parameter | ka_t2m | Kinetic model parameter | Locally identifiable |
| Parameter | ka_tf7a10a | Kinetic model parameter | Locally identifiable |
| Parameter | kap_b2a | Kinetic model parameter | Locally identifiable |
| Parameter | kap_bap | Kinetic model parameter | Locally identifiable |
| Parameter | kd_5a | Kinetic model parameter | Locally identifiable |
| Parameter | kd_at3 | Kinetic model parameter | Locally identifiable |
| Parameter | kd_at3_il6 | Kinetic model parameter | Locally identifiable |
| Parameter | kd_il1b | Kinetic model parameter | Locally identifiable |
| Parameter | kd_il6 | Kinetic model parameter | Locally identifiable |
| Parameter | kd_tf | Kinetic model parameter | Locally identifiable |
| Parameter | kd_tfpi | Kinetic model parameter | Locally identifiable |
| Parameter | kd_tfpi_tnfa | Kinetic model parameter | Locally identifiable |
| Parameter | kd_tnfa | Kinetic model parameter | Locally identifiable |
| Parameter | kda_ec | Kinetic model parameter | Locally identifiable |
| Parameter | kda_mo | Kinetic model parameter | Locally identifiable |
| Parameter | kde_9a_8a_10 | Kinetic model parameter | Locally identifiable |
| Parameter | km2a_2a | Kinetic model parameter | Locally identifiable |
| Parameter | km_22il6 | Kinetic model parameter | Locally identifiable |
| Parameter | km_22t | Kinetic model parameter | Locally identifiable |
| Parameter | km_a1b2a | Kinetic model parameter | Locally identifiable |
| Parameter | koff_10a_5a | Kinetic model parameter | Locally identifiable |
| Parameter | koff_10a_tfpi | Kinetic model parameter | Locally identifiable |
| Parameter | koff_5a10a_2 | Kinetic model parameter | Locally identifiable |
| Parameter | koff_7a_10 | Kinetic model parameter | Locally identifiable |
| Parameter | koff_7a_10a | Kinetic model parameter | Locally identifiable |
| Parameter | koff_7a_9 | Kinetic model parameter | Locally identifiable |
| Parameter | koff_8a | Kinetic model parameter | Locally identifiable |
| Parameter | koff_9a8a_10 | Kinetic model parameter | Locally identifiable |
| Parameter | koff_9a_8a | Kinetic model parameter | Locally identifiable |
| Parameter | koff_tf_vii | Kinetic model parameter | Locally identifiable |
| Parameter | koff_tf_viia | Kinetic model parameter | Locally identifiable |
| Parameter | koff_tfpi_10a_tf_7a | Kinetic model parameter | Locally identifiable |
| Parameter | kon_10a_5a | Kinetic model parameter | Locally identifiable |
| Parameter | kon_10a_tfpi | Kinetic model parameter | Locally identifiable |
| Parameter | kon_10atfpi_tf7a | Kinetic model parameter | Locally identifiable |
| Parameter | kon_5a10a_2 | Kinetic model parameter | Locally identifiable |
| Parameter | kon_7a_10 | Kinetic model parameter | Locally identifiable |
| Parameter | kon_7a_10a | Kinetic model parameter | Locally identifiable |
| Parameter | kon_7a_9 | Kinetic model parameter | Locally identifiable |
| Parameter | kon_8a1_8a2 | Kinetic model parameter | Locally identifiable |
| Parameter | kon_9a8a_10 | Kinetic model parameter | Locally identifiable |
| Parameter | kon_9a_8a | Kinetic model parameter | Locally identifiable |
| Parameter | kon_at3_10a | Kinetic model parameter | Locally identifiable |
| Parameter | kon_at3_2a | Kinetic model parameter | Locally identifiable |
| Parameter | kon_at3_2am | Kinetic model parameter | Locally identifiable |
| Parameter | kon_at3_9a | Kinetic model parameter | Locally identifiable |
| Parameter | kon_at3_tf_7a | Kinetic model parameter | Locally identifiable |
| Parameter | kon_tf_vii | Kinetic model parameter | Locally identifiable |
| Parameter | kon_tf_viia | Kinetic model parameter | Locally identifiable |
| Parameter | kon_tfpi_10a_tf_7a | Kinetic model parameter | Locally identifiable |
| Parameter | kt_22il6 | Kinetic model parameter | Locally identifiable |
| Parameter | kt_22t | Kinetic model parameter | Locally identifiable |
| Parameter | kt_at3 | Kinetic model parameter | Locally identifiable |
| Parameter | kt_epcr | Kinetic model parameter | Locally identifiable |
| Parameter | kt_il1b | Kinetic model parameter | Locally identifiable |
| Parameter | kt_il6 | Kinetic model parameter | Locally identifiable |
| Parameter | kt_plat | Kinetic model parameter | Locally identifiable |
| Parameter | kt_tf | Kinetic model parameter | Locally identifiable |
| Parameter | kt_tf_ec | Kinetic model parameter | Locally identifiable |
| Parameter | kt_tf_mo | Kinetic model parameter | Locally identifiable |
| Parameter | kt_tfpi | Kinetic model parameter | Locally identifiable |
| Parameter | kt_tnfa | Kinetic model parameter | Locally identifiable |

**Supplementary Figure List**

Supplementary Figure S1. PRCC-based parameter sensitivity analysis for endogenous thrombin potential (ETP).

Supplementary Figure S2. PRCC-based parameter sensitivity analysis for lag time (LT).

Supplementary Figure S3. PRCC-based parameter sensitivity analysis for peak thrombin generation (PEAK).

Supplementary Figure S4. PRCC-based parameter sensitivity analysis for time to peak thrombin (TTP).

Supplementary Figure S5. Pre-calibration comparison of peak thrombin across four disease conditions.

Supplementary Figure S6. Pre-calibration comparison of time to peak across four disease conditions.

Supplementary Figure S7. Pre-calibration comparison of lag time across four disease conditions.

Abbreviations: PRCC, partial rank correlation coefficient; LHS, Latin hypercube sampling; PEAK, peak thrombin generation; TTP, time to peak thrombin; ETP, endogenous thrombin potential; LT, lag time; TG, thrombin generation; SCD, sickle cell disease; T2DM, type 2 diabetes mellitus.

**Supplementary Figure S1.**

*PRCC-based parameter sensitivity analysis for endogenous thrombin potential (ETP).*

**Supplementary Figure S1.** Horizontal bars show partial rank correlation coefficient (PRCC) values between sampled model parameters and ETP, calculated as the area under the Phase 2 thrombin generation curve. Positive values indicate parameters associated with higher ETP, whereas negative values indicate parameters associated with lower ETP. Parameters are ordered by absolute PRCC magnitude.

**Supplementary Figure S2.**

*PRCC-based parameter sensitivity analysis for lag time (LT).*

**Supplementary Figure S2.** Horizontal bars show PRCC values between sampled model parameters and LT, defined as the time required for thrombin to reach one-sixth of peak thrombin. Positive values indicate parameters associated with delayed thrombin initiation, whereas negative values indicate parameters associated with shorter lag time. Parameters are ordered by absolute PRCC magnitude.

**Supplementary Figure S3.**

*PRCC-based parameter sensitivity analysis for peak thrombin generation (PEAK).*

**Supplementary Figure S3.** Horizontal bars show PRCC values between sampled model parameters and the maximum thrombin concentration during Phase 2 thrombin generation. Positive values indicate parameters associated with higher peak thrombin, whereas negative values indicate parameters associated with lower peak thrombin. Parameters are ordered by absolute PRCC magnitude.

**Supplementary Figure S4.**

*PRCC-based parameter sensitivity analysis for time to peak thrombin (TTP).*

**Supplementary Figure S4.** Horizontal bars show PRCC values between sampled model parameters and the time required to reach maximum thrombin concentration during Phase 2 thrombin generation. Positive values indicate parameters associated with longer TTP, whereas negative values indicate parameters associated with faster peak generation. Parameters are ordered by absolute PRCC magnitude.

**Supplementary Figure S5.**

*Pre-calibration comparison of peak thrombin across four disease conditions.*

**Supplementary Figure S5.** Comparison of calibrated simulation, experimental, and uncalibrated virtual patient peak thrombin values across hemophilia A, COVID-19, sickle cell disease (SCD), and type 2 diabetes mellitus (T2DM). The uncalibrated results represent the initial virtual patient sampling ranges before tuning to match experimental peak thrombin values. This comparison illustrates the discrepancy between the pre-calibration sampling ranges and the experimental data that motivated the peak-based calibration step.

**Supplementary Figure S6.**

*Pre-calibration comparison of time to peak across four disease conditions.*

**Supplementary Figure S6.** Comparison of calibrated simulation, experimental, and uncalibrated virtual patient time-to-peak values across hemophilia A, COVID-19, SCD, and T2DM. The uncalibrated results show model behavior before the peak-based tuning step. After calibration using peak thrombin values, time to peak was used as an independent validation metric for the disease-specific sampling ranges.

**Supplementary Figure S7.**

*Pre-calibration comparison of lag time across four disease conditions.*

**Supplementary Figure S7.** Comparison of calibrated simulation, experimental, and uncalibrated virtual patient lag time values across hemophilia A, COVID-19, SCD, and T2DM. The uncalibrated results provide the corresponding pre-calibration reference. Lag time was not directly used for fitting and was instead used to assess the validity of the calibrated sampling ranges.
